# Tuberculosis among correctional facility workers: A systematic review and meta-analysis

**DOI:** 10.1371/journal.pone.0207400

**Published:** 2018-11-15

**Authors:** Micheli Luize Grenzel, Antonio José Grande, Anamaria Mello Miranda Paniago, Mauricio Antonio Pompilio, Sandra Maria do Valle Leone de Oliveira, Anete Trajman

**Affiliations:** 1 Mestrado Profissional em Saúde da Familia, Universidade Federal de Mato Grosso do Sul Campo Grande, Brazil; 2 Faculdade de Medicina, Universidade Estadual de Mato Grosso do Sul, Campo Grande, Brazil; 3 Faculdade de Medicina, Universidade Federal de Mato Grosso do Sul Campo, Grande, Brazil; 4 Universidade Federal do Rio de Janeiro, Rio de Janeiro, Brazil; 5 McGill University, Montreal, Quebec, Canada; The University of Warwick, UNITED KINGDOM

## Abstract

**Introduction:**

Prison inmates can transmit tuberculosis, including drug-resistant strains, to correctional facility workers and the community. In this systematic literature review, we investigated the magnitude of active and latent tuberculosis infection (LTBI) and associated risk factors among correctional facility workers.

**Methods:**

We searched MEDLINE, EMBASE, LILACS, Cochrane CENTRAL, ISI Web of Science, CINAHL, and SCOPUS databases (January 1, 1989–December 31, 2017) for studies with the MeSH terms “prison” (and similar) AND “tuberculosis”, without language restriction. We searched for gray literature in Google Scholar and conference proceedings. Stratified analyses according to tuberculosis burden were performed.

**Results:**

Of the 974 titles identified, 15 (nine good, six fair quality) fulfilled the inclusion criteria (110,393 correctional facility workers; six countries; 82,668 active tuberculosis; 110,192 LTBI). Pooled LTBI prevalence and incidence rates were 26% (12–42, I^2^ = 99.0%) and 2% (1–3, I^2^ = 98.6%), respectively. LTBI prevalence reached 44% (12–79, I^2^ = 99.0%) in high-burden countries. Active tuberculosis was reported only in low-burden countries (incidence range, 0.61–450/10,000 correctional facility workers/year). LTBI-associated risk factors included job duration, older age, country of birth, current tobacco smoking, reported contact with prisoners, and BCG vaccination.

**Conclusion:**

Despite the risk of bias and high heterogeneity, LTBI was found to be prevalent in correctional facility workers, mainly in high-burden countries. LTBI risk factors suggest both occupational and community exposure. Active tuberculosis occurrence in low-burden countries suggests higher vulnerability from recent infection among correctional facility workers in these countries. Systematic surveillance and infection control measures are necessary to protect these highly vulnerable workers.

## Introduction

Tuberculosis (TB) is one of the leading causes of morbidity and mortality from infectious diseases worldwide, with 10.4 million new cases and 1.3 million deaths in 2016 [1]. In particular, TB management has become increasingly challenging in inmates of correctional facilities, wherein TB prevalence can be as high as 1,913/100,000 population, with incidence of up to 70/100,000 population/year [2]. The prevalence of latent TB infection (LTBI) among inmates can increase by approximately 5% every year [3], suggesting that there is a high risk of transmission in prisons. Occupational exposure was responsible for recently acquired LTBI in one-third of New York State prison employees [4]. In a TB outbreak in Madrid, Spain, 23% of all cases, including 38 inmates and five employees, were caused by the same strain, according to molecular epidemiological analyses [5].

TB in inmates may also be responsible for transmission to the community. Up to 54% of *Mycobacterium tuberculosis* strains in the community are similar to those found in prisons [6]. Similarly, TB transmission to correctional facility workers (CFW) has also been documented [7–21]. The magnitude of the epidemics in this population, however, varies significantly. While data on burden of TB among inmates is available, data on burden among CFW are only available from individual studies. Moreover, since longitudinal studies are time consuming, labor intensive and expensive, prevalence studies are more likely to be conducted in resource-constrained settings and provide information on LTBI burden. A systematic review on the current burden of LTBI among CFW in high- and low-burden countries would provide evidence of existing occupational risk of infection in prison facilities settings, raise awareness among CFW to adopt and practice necessary infection control measures, and guide policy makers to explore and implement necessary prevention and control measures to reduce disease burden. Because TB is a preventable disease, and prevention is one of the cornerstones of the END TB Strategy [22], it is important to identify populations at high risk for recent LTBI and active TB. Therefore, we conducted a systematic review and meta-analysis to estimate the pooled prevalence and incidence of LTBI and active TB among CFW, as well as their associated risk factors.

## Materials and methods

The study protocol was registered at the International Prospective Register of Systematic Reviews–CRD42016048858. Ethical approval was not necessary, since all data are publicly available. The current report follows the recommendations of the *Preferred Reporting Items for Systematic Reviews and Meta-Analyses* (PRISMA) [23].

### Search strategy

We searched the MEDLINE (through PubMed), EMBASE (through Elsevier), LILACS (through BVS), Cochrane Central Register of Controlled Trials (CENTRAL), ISI Web of Science, CINAHL (through EBSCO), and SCOPUS databases for studies published between January 1, 1989 and December 31, 2017, without language restriction. We searched for gray literature in Google Scholar and congress abstract books. We also searched for relevant studies in the reference lists of articles included in the review. MeSH terms were used for the searches were “tuberculosis” and “prison” (and its synonyms). The search strategy was adapted for each database and is detailed in the supplement material (S1 Table).

### Study selection, inclusion criteria, and data extraction

Identified titles were imported to *EndNote online*, and duplicated studies were removed. The remaining titles were independently reviewed by two authors (MLG and SMVLO), who selected abstracts from articles, with no language restriction. The same authors independently selected full texts from the abstract list. Divergence was resolved by consensus. Observational (including cross-sectional, retrospective, and longitudinal) or experimental studies were eligible, regardless of the population size, if they tested CFW in any sector (security, administration, or healthcare workers). Studies that considered bacteriologically confirmed or clinical-radiological diagnoses of active TB were included. For LTBI diagnosis, we included studies that used either the tuberculin skin test (TST) or interferon-gamma release assays (IGRA). Cut-off values for prevalence and TST conversion were those considered by authors. We excluded studies in which more than one population was described and in which it was not possible to extract or calculate the indicators of interest. We included studies that reported prevalence/incidence rates or allowed the calculation of these variables by providing the necessary data.

### Data pooling and statistical analyses

Pooled prevalence and incidence of active TB and LTBI, and their 95% confidence intervals (CI) were calculated with STATA software (STATA/SE 12.1, StataCorp, College Station, TX, USA) using random effects models. I^2^ values of 25–49%, 50–74%, and ≥75% were considered to represent low, moderate, and high levels of heterogeneity [24], respectively.

Sources of heterogeneity were explored using sensitivity analyses. Analyses were performed by removing studies with less than 100 participants, and subgroup analyses were conducted according to diagnostic criteria and the country’s TB burden [1].

### Quality of study methods

For quality evaluation, we used the *Quality Assessment Tool for Observational Cohort and Cross-sectional Studies* of the National Heart, Lung and Blood Institute [25], which evaluates the internal validity of studies. Each study was assessed for 14 criteria: the research question or objective, the study population, the participation rate of eligible persons, individuals selected or recruited from the same or similar populations, sample size justification, exposure(s) of interest measured before outcome, sufficient deadline, different levels of exposure to outcome, exposure measurement (independent variables) clearly defined, blinded as to the exposure status of participants, follow-up, main potential confounders measured and statistically adjusted. Studies were classified as good, fair, or poor if they fulfilled ≥12, 5–11, and < 5 criteria, respectively.

### Publication bias

Publication bias and small study effect were evaluated using funnel plot analysis [24] when ≥10 studies were included.

## Results

We identified 3028 titles: 3019 from the databases and nine through additional searches. After removing duplicates, 974 titles remained, of which 948 abstracts were excluded because they did not analyze TB or LTBI among CFW. Of the 26 abstracts retained for full text reading, 11 studies were further excluded: three could not be found even after contacting the author(s) [26–28] and eight did not allow calculation of prevalence or incidence in the population of interest [29–36]. Thus, 15 studies were included in this review and 14 in the meta-analysis (S1 Fig).

### Study and population characteristics

The 15 studies were published between 1989 and 2017 (latest data collection in 2015) and were conducted in six countries: Australia, Brazil, Canada, Malaysia, Malawi, and the United States of America (USA) [7–21]. Fourteen evaluated LTBI: 11 reported LTBI prevalence [8–13, 15, 16, 19–21] and eight [8, 11, 12, 14–18] reported LTBI incidence. All were included in the meta-analyses. All but one used TST to diagnose LTBI [10]. Details regarding TST technique and cut-off points are displayed in Table 1. Four studies also evaluated the incidence of active TB [7, 12, 14, 18]. Diagnostic criteria for active TB included sputum smear microscopy [7, 14], culture [12, 14], and chest X-ray [14]. Only one study did not report diagnostic criteria [18]. Mean age of prison workers ranged from 18 to 64 years. The study subjects were predominantly male among the eight studies that reported sex [9, 10, 12, 13, 16, 17, 20, 21]. Bacillus Calmette-Guérin (BCG) vaccination was reported in five studies (Table 1) [9, 10, 16, 19, 20].

**Table 1:** Characteristics of studies.

Author (year) [ref.]	Country	Year of data collection	Study design	Participants	Location	BCG %, (n/N)	Male sex (%, n/N)	Mean age or range	Tuberculin, cut-off value and time of reading
Busatto et al. (2017) [21]	Brazil	2015	Cross-sectional	Health care, security staff, and administrative staff	Four prisons in two regions of Rio Grande do Sul	NA	58.1% (66/114)	30–39	NA
Oliveira et al. (2017) [10]	Brazil	2013	Cross-sectional	Health care, security staff, and administrative staff	Four prisons in Mato Grosso do Sul	84.7% (161/190)	49.5% (94/190)	27–64	PPD RT-23 (0.1 ml);≤ 10 mm;48–72 h
Youakim (2016) [14]	Canada	1999–2008	Retrospective cohort	Corrections officer, police officer, and sheriff	British Columbia (Statistics Canada censuses)	NA	NA	NA	NA
Al-Darraji et al. (2015) [20]	Malaysia	2011	Cross-sectional	Correctional officers, healthcare personnel, and administrative staff.	Kajang Prison (the largest prison in Malaysia).	98.1% (412/420)	88.8% (373/420)	NA	PPD RT-23; ≤ 10 mm;48–72 h
Nogueira et al. (2011) [13]	Brazil	2008	Cross-sectional	Health care and security staff	Two prisons in Guarulhos (State of São Paulo)	NA	81.9% (227/277)	18–50	PPD RT- 23; ≤ 10 mm; after 72 h
Binswanger et al. (2010) [18]	USA	2006–2007	Retrospective cohort	Correctional officers	Jails from 49 states	NA	NA	NA	NA
Mitchell et al. (2005) [16]	USA	1999–2000	Prospective cohort	Correctional healthcare workers	Departments of corrections in Rhode Island, Maryland and Texas	9.6% (39/408)	25.2% (103/408)	44	Tubersol PPD 5 TU;≤ 10 mm;48–72 h.
Kachisi et al. (2002) [7]	Malawi	2000	Cross-sectional	Prison staff	Four prisons in Zomba	NA	NA	30–55	NA
Cooper-Arnold et al. (1999) [11]	USA	1993–1995	Cross-sectional	Deputy sheriffs	Prisons in Connecticut State	NA	NA	25–45	Applisol PPD 5 TU;≤ 10 mm;48–72 h
Jones et al. (1999) [12]	USA	1996–1997	Retrospective cohort	Jail staff	Memphis criminal justice center	NA	44% (348/790)	34	PPD (5TU) ≤ 10 mm;48–72 h (Laboratory of PPD not provided in the paper)
MacIntyre et al. (1999) [19]	Australia	1997	Cross-sectional	Staff in prison	A prison in the state of Victoria	70.4% (38/54)	NA	33–42	PPD RT-23; ≤10 mm without BCG,≤15 mm with BCG.
Jochem et al. (1997) [9]	Canada	1995	Cross-sectional	Prison guard and others	Prison for women in Montreal	49% (50/102)	23.5% (24/102)	37.5	PPD-T bioequivalent to PPD-S (Connaught, Toronto), 5 TU; ≤ 10 mm; 48–72 h
Steenland et al. (1997) [17]	USA	1991–1992	Prospective cohort	Corrections officers, social workers, teachers, medical personnel, and maintenance workers	New York Department of Corrections	NA	80% (19,590/ 24,487)	40	PPD (Laboratory of PPD not provided in the paper); ≤10 mm; 48–72h
Centers for Disease Control and Prevention (1993) [15]	USA	1987–1991	Prospective cohort	Employed infirmary physicians and nurses	One prison in California	NA	NA	NA	PPD (Laboratory of PPD not provided in the paper); ≤10 mm; 48–72h
Spencer e Morton (1989) [8]	USA	1986–1987	Prospective cohort	Employees of correctional facilities	Seven correctional facilities in New Mexico	NA	NA	NA	PPD 5 TU; ≤10 mm; 48–72 h

BCG, Bacillus Calmette-Guérin; NA, non-available; NR, not-performed; PPD, purified protein derivative; TU, tuberculin units.

## Study findings

### Prevalence of LTBI

Overall, 110,192 CFW were evaluated for LTBI in five countries between 1986 and 2015 (Table 2). Pooled LTBI prevalence was 26% (95% CI = 12–42%, S2 Fig). Heterogeneity was high (I^2^ = 99.0%): prevalence of LTBI ranged from 6% in Australia [19] to 81% in Malaysia (S3 Fig) [20]. Exclusion of studies with less than 100 participants did not change the results significantly: pooled LTBI prevalence was 29% (95% CI = 14–47%), and the heterogeneity remained high (I^2^ = 99.0%). Pooled prevalence was 16% (95% CI = 10–22%, I^2^ = 93.3%) in countries with low TB burden, compared with 44% (95% CI = 12–79%, I^2^ = 99.0%) in countries with high TB burden (S4 Fig).

**Table 2:** The prevalence of latent tuberculosis infection and incidence of active tuberculosis, and the latent tuberculosis infection.

Study	Latent tuberculosis infection		Incidence of active tuberculosis rate/10,000 year, (n/N)
	Prevalence	Incidence	
	(%, n/N)	(%, n/N)	
**Kachisi et al. (2002) [7]**	-	-	450/1 y, (9/201)
**Spencer and Morton (1989) [8]**	10% (129/1,323)	1% (10/1,184)	-
**Jochem et al. (1997) [9]**	32% (33/102)	-	-
**Oliveira et al. (2017) [10]**	12% (22/187)	-	-
**Cooper-Arnold et al. (1999) [11]**	9% (48/539)	6% (22/377)	-
**Jones et al. (1999) [12]**	21% (147/706)	1% (8/546)	21/3 y, (5/790)
**Nogueira et al. (2011) [13]**	57% (142/248)	-	-
**Youakim (2016) [14]**	-	12% (8/67)	15/10 y, (1/67)
**Centers for Disease Control and Prevention (1993) [15]**	28.6% (6/21)	20.0% (2/10)	-
**Mitchell et al. (2005) [16]**	17.7% (68/385)	1.3% (3/231)	-
**Steenland et al. (1997) [17]**	-	1.9% (466/24,487)	-
**Binswanger et al. (2010) [18]**	-	0.4% (322/81,610)	0.61/1 y, (5/81,610)
**MacIntyre et al. (1999) [19]**	5.6% (3/54)	-	-
**Al-Darraji et al. (2015) [20]**	81.0% (340/420)	-	-
**Busatto et al. (2017) [21]**	28% (12/43)	-	-

Study [14] reports the number of LTBI in 10 years and study [15] in four years.

### Incidence of LTBI

LTBI incidence was reported only in studies (n = 8) conducted in low-burden countries [8, 11, 12, 14–18]. Pooled LTBI incidence in 108,512 CFW was 2% (95% CI = 1–3%, S5 Fig), with high heterogeneity (I^2^ = 98.6%). Similar to the prevalence findings, removal of studies with less than 100 participants and of one study [14, 15] that tested conversion 4 years after the first TST [15] did not change the results: pooled incidence remained 2% (95% CI = 1–3%, I^2^ = 98.9%).

### Active TB

Overall, 82,668 CFW were evaluated for active TB in three countries [7, 12, 14, 18] between 1999 and 2016. Incidence of active TB ranged from 0.61 to 450/10,000 CFW/year. We did not perform meta-analyses, since the time of follow up was 1, 3, and 10 years.

### Risk factors

We heterogeneously analyzed risk factors for prevalence and incidence of LTBI in seven studies that controlled for confounding by using multivariate regression models (Table 3). Older age, BCG vaccination after infancy, history of contact with prisoners, work in correctional facilities for more than one year, permanence in endemic countries for more than three months, place of birth, and current smoking status were found to be associated with LTBI. Risk factors for active TB were not reported in any of the four studies.

**Table 3:** Significant risk/associated factors for positive tuberculin skin test results after multivariate analysis.

Variables	Factors	
	Associated [ref.]	Risk [ref.]
Male sex	-	[16]
Old age	[11], [19]	-
Total duration of work in the correctional system > 12 months	[9], [11], [20], [21]	-
History of Bacillus Calmette-Guérin vaccination	[9]	[16]
Reported history of contact with prisoners	[13]	-
Current tobacco smoking	[20]	-
Country of birth	[19]	[16]
Travel to tuberculosis-endemic countries for >3 months	[9]	-
Region of country	[21]	-

Associated factors extracted from cross-sectional studies and risk factors extracted from cohort studies.

### Quality of studies: Risk of bias

Nine studies were considered to be of good quality [7, 9, 11–13, 15, 16, 19, 20], and six were considered to be of fair quality S2 Table [8, 10, 14, 17, 18, 21]. Limitations of studies included lack of justification of sample selection [7–11, 13–19, 21], poor reliability of the measure of exposure (self-reporting), and >20% of the initial population being lost to follow up [21], The main limitation, however, was the absence of adjustment of risk factors for confounding variables [7, 8, 10, 12, 14, 15, 17, 18].

### Publication bias

The funnel plot for the included studies was not symmetrical (S6 Fig). Most studies are outside the 99.8% limit. Six studies [8, 10, 14, 17, 18, 21] contribute to the asymmetry, with smaller standard error and greater effect size, suggesting a risk of publication bias.

## Discussion

TB transmission among inmates in correctional facilities is well reported, and represents a challenge for TB control [37]. Prisons and jails are frequently overcrowded, and the environmental conditions facilitate transmission [38]. The current study shows that the high risk of TB transmission is not restricted to inmates. The occurrence of LTBI is frequent among CFW, regardless of whether they worked in administrative, security, or healthcare services. Prevalence and incidence of LTBI are especially high in countries with high TB burden, despite the high heterogeneity among studies. Heterogeneity may be partially explained by the settings (TB burden, BCG vaccination policies), but methodological differences in sample selection and outcome definition (different cut-off values for TST) may also have played an important role.

Previous evidence suggests that TB should be considered an occupational disease in CFW [4, 5, 20]. Our study supports this point of view, although it is difficult to prove that transmission occurred within the correctional facilities. There are more studies on LTBI than on active TB and, unlike active TB, epidemiologic molecular evidence cannot be obtained to establish the source of LTBI transmission. However, indirect evidence suggests occupational transmission. Firstly, the magnitude of LTBI is similar to that reported in healthcare workers, a population known to have high work-related exposure [39]. Moreover, many of the reported LTBI risk factors for CFW are the same as those for healthcare workers [40, 41]. In addition, molecular studies have shown that inmates are a source of TB transmission in both the correctional facilities and the community [6]. Finally, in our review, the duration of work in correctional facilities was strongly and significantly associated with higher risk for LTBI in low-burden countries [9, 11, 20], whereas duration of contact with inmates was the main factor associated with LTBI in high-burden countries. These findings corroborate the plausibility of the occupational nature of the transmission. Nevertheless, a significant correlation was also found between TB risk and non-occupational variables, such as older age, place of birth, trips longer than three months and BCG after infancy in low-burden countries, and smoking [9, 11, 16, 19, 20] and region of the country in high-burden countries [21], suggesting that community exposure–as well as the BCG effect on TST results–has an effect in this setting.

The included studies had some limitations, although the methodological quality was considered good in nine studies. Most studies were cross-sectional, and therefore, evaluated the prevalence of LTBI; however, data on the incidence of LTBI would provide better evidence for occupational exposure, or at least, for recent infection. TST (and interferon-γ release assays) cannot distinguish between recent and remote infection [42]; thus, it is not possible to infer that infection occurred in the correctional facilities. Data stratified by occupation and exposure were not provided, precluding meta-regression and stratified analyses by level of exposure. Finally, most studies were conducted more than 17 years ago, and may not reflect the current situation.

Our review also presents limitations. The funnel plot suggests that we might have overestimated the prevalence of LTBI, since a larger effect size is suggested. However, the study has many strengths. We searched nine comprehensive databases and the gray literature, with no language restriction, and we had access to most of the literature.

In summary, employees of correctional facilities are at risk for TB. These findings emphasize the need for infection control measures in such high-risk settings. Close surveillance and timely treatment when necessary are recommended. More studies are required to elucidate the risk factors for TB in this setting.

## Supporting information

S1 Checklist This is checklist PRISMA. (DOC)

S1 Table This is the S1 Table search strategy. (DOCX)

S2 Table This is the S2 Table study quality assessment. (DOCX)

S1 Fig This is the S1 Fig flowchart of study selection. (DOC)

S2 Fig This is the S2 Fig forest plot showing study-specific and pooled estimates of the prevalence of latent tuberculosis infection. (DOCX)

S3 Fig This is S3 Fig forest plot showing country-specific pooled estimates of the prevalence of latent tuberculosis infection. (DOCX)

S4 Fig This is S4 Fig forest plot showing pooled estimates of the prevalence of latent tuberculosis infection according to low and high burden countries. (DOCX)

S5 Fig This is S5 Fig forest plot showing study-specific and pooled estimates of the incidence of latent tuberculosis infection. (DOCX)

S6 Fig This is S6 Fig funnel plot showing pooled estimates of the prevalence of latent tuberculosis infection. (DOCX)
