# Supplementary material for: Tuberculosis among correctional facility workers: A systematic review and meta-analysis
Source: PLoS One. 2018 Nov 15;13(11):e0207400. doi: 10.1371/journal.pone.0207400 (PMC6237373; doi:10.1371/journal.pone.0207400)
Supplement: S1 Table — (DOCX) [file pone.0207400.s002.docx]

**S1 Table Search strategy**

| **MEDLINE (through PubMed)** | ("Tuberculosis"[Mesh] OR ("tuberculosis"[MeSH Terms] OR "tuberculosis"[All Fields] OR "tuberculoses"[All Fields]) OR ("tuberculosis"[MeSHTerms] OR "tuberculosis"[All Fields] OR ("koch’s"[All Fields] AND "disease"[All Fields])) OR ("tuberculosis"[MeSH Terms] OR "tuberculosis"[All Fields] OR ("disease"[All Fields] AND "koch’s"[All Fields])) OR ("tuberculosis"[MeSH Terms] OR "tuberculosis"[All Fields] OR ("koch's"[All Fields] AND "disease"[All Fields]) OR "koch's disease"[All Fields]) OR ("tuberculosis"[MeSH Terms] OR "tuberculosis"[All Fields] OR ("disease"[All Fields] AND "koch's"[All Fields])) OR ("tuberculosis"[MeSH Terms] OR "tuberculosis"[All Fields] OR ("koch’s"[All Fields] AND "disease"[All Fields]) OR "koch’s disease"[All Fields]))AND ("Prisons"[Mesh] OR ("prisons"[MeSH Terms] OR "prisons"[All Fields] OR "prison"[All Fields])). |
| --- | --- |
| **EMBASE (through Elsevier)** | 1. exp tuberculosis/ 2. exp prison/ 3. 1 and 2 |
|  |  |
|  |  |
|  |  |
