# Supplementary material for: Tuberculosis among correctional facility workers: A systematic review and meta-analysis
Source: PLoS One. 2018 Nov 15;13(11):e0207400. doi: 10.1371/journal.pone.0207400 (PMC6237373; doi:10.1371/journal.pone.0207400)
Supplement: S1 Fig — (DOC) [file pone.0207400.s004.doc]

**S1Fig Flowchart of study selection**

**Screening**

**Included**

**Eligibility**

**Identification**

Records identified through database searching
(n = 3019)

Additional records identified through other sources
(n = 09)

Records after excluding duplicates
(n = 974)

Records screened
(n = 26)

Full-text articles assessed for eligibility
(n = 26)

Studies included in the qualitative synthesis
(n = 15)

Studies included in quantitative synthesis (meta-analysis).
(n = 14)

Records excluded
(n = 948)

Full-text articles excluded
(n = 11)Three full-text articles were not accessible and eight did not include indicators for the population of interest.
