# Supplementary material for: Tuberculosis among correctional facility workers: A systematic review and meta-analysis
Source: PLoS One. 2018 Nov 15;13(11):e0207400. doi: 10.1371/journal.pone.0207400 (PMC6237373; doi:10.1371/journal.pone.0207400)
Supplement: S4 Fig — (DOCX) [file pone.0207400.s007.docx]

**S4 Fig Forest plot showing pooled estimates of the prevalence of latent tuberculosis infection according to low and high burden countries**

Note: ES, prevalence/proportion estimated; CI, confidence interval.
